# Violent experiences and neighbourhoods during adolescence: understanding and mitigating the association with mental health at the transition to adulthood in a longitudinal cohort study

**DOI:** 10.1007/s00127-022-02343-6

**Published:** 2022-08-09

**Authors:** Rachel M. Latham, Louise Arseneault, Bianca Alexandrescu, Saffron Baldoza, Alysha Carter, Terrie E. Moffitt, Joanne B. Newbury, Helen L. Fisher

**Affiliations:** 1 King’s College London, Social, Genetic and Developmental Psychiatry Centre, Institute of Psychiatry, Psychology and Neuroscience, 16 De Crespigny Park, London, SE5 8AF UK; 2 ESRC Centre for Society and Mental Health, King’s College London, London, UK; 3 Independent Researcher, Essex, UK; 4 Independent Researcher, Buckinghamshire, UK; 5 Independent Researcher, Hertfordshire, UK; 6 Department of Psychology and Neuroscience, Duke University, Durham, NC USA; 7 Psychiatry and Behavioral Sciences, Duke University, Durham, NC USA; 8 Center for Genomic and Computational Biology, Duke University, Durham, NC USA; 9 Population Health Sciences: Bristol Medical School, University of Bristol, Bristol, UK

**Keywords:** Mental health, Neighbourhood characteristics, Protective factors, Resilience, Social support

## Abstract

**Purpose:**

Violence occurs at multiple ecological levels and can harm mental health. However, studies of adolescents’ experience of violence have often ignored the community context of violence, and vice versa. We examined how personal experience of severe physical violence and living in areas with high levels of neighbourhood disorder during adolescence combine to associate with mental health at the transition to adulthood and which factors mitigate this.

**Method:**

Data were from the Environmental Risk Longitudinal Twin Study, a nationally representative birth cohort of 2232 British twins. Participants’ experience of severe physical violence during adolescence and past-year symptoms of psychiatric disorder were assessed via interviews at age 18. Neighbourhood disorder was reported by residents when participants were aged 13–14. Potential protective factors of maternal warmth, sibling warmth, IQ, and family socio-economic status were assessed during childhood, and perceived social support at age 18.

**Results:**

Personal experience of severe physical violence during adolescence was associated with elevated odds of age-18 psychiatric disorder regardless of neighbourhood disorder exposure. Cumulative effects of exposure to both were evident for internalising and thought disorder, but not externalising disorder. For adolescents exposed to severe physical violence only, higher levels of perceived social support (including from family and friends) were associated with lower odds of psychiatric disorder. For those who also lived in areas with high neighbourhood disorder, only family support mitigated their risk.

**Conclusion:**

Increasing support or boosting adolescents’ perceptions of their existing support network may be effective in promoting their mental health following violence exposure.

**Supplementary Information:**

The online version contains supplementary material available at 10.1007/s00127-022-02343-6.

An estimated 1.6 million people worldwide lose their lives to violence each year, while many more suffer life-altering consequences including mental health problems [1]. Violence is a complex phenomenon; it can take many different forms and occur at different—as well as multiple—ecological levels. At the inter-personal level, violence may be experienced personally (e.g., sexual and physical victimisation) through interactions with peers, family members, and others in the wider environment. At the community level, individuals may live in neighbourhoods where there is violence that is not directed at them or witnessed personally. This includes physical and social signs of violence, threat, and danger, collectively referred to as neighbourhood disorder [2].

Adolescence is a peak age for experiencing several types of violence including physical assault, sexual victimisation, and family violence [3]. It is also when youth begin to spend more time unsupervised in the community, potentially exposing them to a wider range of violence and facilitating a greater awareness of neighbourhood disorder. At the same time, adolescence and the transition to adulthood is a high-risk period for the onset of a variety of common psychiatric disorders [4, 5] that for many will signal the start of recurrent mental illness throughout adulthood [6]. Indeed, around 75% of adult mental health disorders will have onset by the age of 18 [7]. A better understanding of how personal experiences of violence and high levels of neighbourhood disorder during adolescence are associated with the development of mental health diagnoses could inform early targeted intervention and prevention.

Evidence consistently shows that personal experiences of violence, especially during childhood and adolescence, elevate the risk for psychiatric disorders [8, 9] including internalising disorders such as depression and anxiety [10, 11], externalising disorders such as attention-deficit hyperactivity disorder (ADHD) [12], and psychosis [13, 14]. Separately, studies of community-level violence have also shown associations with residents’ mental health problems [15–17]. Although research specifically with adolescents is scarce, links between neighbourhood disorder and adolescent psychological distress [18] and psychotic experiences [19] have been evidenced.

However, different types of violence often converge, and therefore, it is also important to consider the combined impact of multiple forms of exposure. From the cumulative stress perspective, the greater the number of risk factors an individual experiences, the greater their risk of suffering from mental health problems [20, 21]. Alternatively, exposure to violence across multiple settings may normalise or desensitise individuals such that one exposure reduces the effects of the other [22]. Individuals who experience one type of violence also commonly experience another type [23, 24] and this poly-victimisation confers even greater risk for mental health problems [9, 25–27]. Violence exposure may also converge across ecological levels; for example, people who live in neighbourhoods with high levels of disorder are more likely to have personal experience of crime [15, 28]. However, unlike poly-victimisation, there has been limited investigation of how multi-level violence exposure during adolescence combines to impact mental health. One study has shown cumulative effects of adverse living conditions (including neighbourhood disorder) and crime victimisation on adolescent psychotic experiences [28]. However, studies of community-level violence and mental health have typically ignored [29] or been conflated [30] with personal experiences of violence, while studies of inter-personal violence often do not consider the social context of violence in which these may take place. Thus, understanding how these two levels of violence exposure operate in the context of one another in relation to mental health represents a significant gap in the literature.

Fortunately, not everyone who is exposed to violence develops mental health problems. For example, in a UK cohort, 40% of young people did not have a psychiatric disorder at age 18 despite experiencing severe childhood victimisation [31]. Understanding what factors protect against poor mental health among adolescents exposed to violence is necessary to inform interventions at the individual, family, and community levels to mitigate its effects. Existing research has primarily focused on factors that are protective following violence that is personally experienced [32], but it remains unknown what factors are protective for adolescents who are also exposed to violence at the community level.

The present study addresses these knowledge gaps using data from the Environmental Risk (E-Risk) Longitudinal Twin Study. We investigate: (i) how the prevalence of psychiatric disorder at age-18 compares between adolescents with personal experience of severe physical violence, those who lived in neighbourhoods with high levels of disorder, and those with no such exposure during adolescence; (ii) whether there is a cumulative effect of having both personal experience of severe physical violence and living in a neighbourhood with high levels of disorder during adolescence on age-18 psychiatric disorder; and (iii) whether supportive relationships (maternal warmth, sibling warmth, and perceived social support), higher IQ, and higher family socio-economic status (SES) protect against the development of psychiatric disorder within those violence-exposed groups who are at elevated risk.

The putative protective factors that we investigate were identified during focus group discussions with a group of young people with lived experience of violence, abuse, and mental health problems (see Latham et al. [33] for details of the focus groups) and then matched to measures available in the E-Risk Study. These factors are also consistent with theoretical accounts of resilience that highlight physical, psychological, and social resources in the environment that can help individuals to sustain their wellbeing in the face of adverse circumstances [34] as well as empirical findings [32, 35, 36]. Involving individuals with lived experience in mental health research ensures that it is relevant, inclusive, and high quality [37, 38]. Accordingly, peer researchers also partnered with the academic research team to help interpret and present the study findings.

## Method

### Sample

Participants were members of the Environmental Risk (E-Risk) Longitudinal Twin Study, which tracks the development of a nationally representative birth cohort of 2232 British children. The sample was drawn from a larger birth register of twins born in England and Wales in 1994–1995 [39]. Full details about the sample are reported elsewhere [40], and in Supplementary Material. Briefly, the E-Risk sample was constructed in 1999–2000 when 1116 families (93% of those eligible) with same-sex 5-year-old twins participated in home-visit assessments. Sex was evenly distributed within zygosity (49% male).

Follow-up home-visits were conducted when the children were aged 7, 10, 12, and 18 years (participation rates were 98%, 96%, 96%, and 93%, respectively). There were 2066 E-Risk participants (47% male) who were assessed at age 18. The average age of the participants at the time of the assessment was 18.4 years (SD = 0.36); all interviews were conducted after the 18th birthday. There were no differences between those who did and did not take part at age 18 in terms of socio-economic status (SES) assessed when the cohort was initially defined (*χ*^2^ = 0.86, *p* = 0.65), age-5 IQ scores (*t* = 0.98, *p* = 0.33), age-5 behavioural (*t* = 0.40, *p* = 0.69) or emotional (*t* = 0.41, *p* = 0.68) problems, or childhood poly-victimisation (*z* = 0.51, *p* = 0.61).

The Joint South London and Maudsley and the Institute of Psychiatry Research Ethics Committee approved each phase of the study. Parents gave informed consent and participants gave assent between 5 and 12 years and then informed consent at age 18.

### Measures

#### Personal experience of severe physical violence during adolescence

At age 18, participants were interviewed face-to-face about exposure to a range of adverse experiences between 12 and 18 years using the Juvenile Victimisation Questionnaire (JVQ) [41, 42] adapted as a clinical interview (see Supplementary Material and Fisher et al. [24] for full details). All information from the JVQ interview was compiled into victimisation dossiers. Using these dossiers, an expert in victimology (HLF) and three other members of the E-Risk team evaluated whether each participant was exposed to any physical violence, whether in the family, by peers, or by people in the wider environment. This “any physical violence” exposure variable was rated on a 6-point scale: 0 = not exposed, then 1–5 for increasing levels of severity (see Supplementary Table S1 for coding detail). The anchor points for these ratings were adapted from the coding system used for the Childhood Experience of Care and Abuse interview (CECA) [43, 44]. Consistent with previous studies using the CECA [43, 45], to index the most severe experiences of violence we dichotomised this variable such that those scoring at the upper end of the severity scale (4–5) were identified as having personal experience of severe violence (coded 1: 24.3% of participants, N = 502).

#### Neighbourhood disorder during adolescence

Neighbourhood disorder (14 items) was assessed via a postal survey sent to residents living alongside E-Risk families when participants were aged 13–14. Survey respondents, who were typically living on the same street or within the same apartment block as the Study participants, reported on various characteristics of their immediate neighbourhood, including levels of neighbourhood disorder. Surveys were returned by an average of 5.18 (*SD* = 2.73) respondents per neighbourhood, and there were at least 2 responses for 95% of neighbourhoods (*N* = 5 601 respondents). Residents were asked whether certain problems affected their neighbourhood, including muggings, assaults, vandalism, graffiti, and deliberate damage to property. Items were coded 0 (‘no, not a problem’), 1 (‘yes, somewhat of a problem’) or 2 (‘yes, a big problem’). Consistent with existing analyses of the E-Risk Study [28, 46–49], items were averaged to create a summary score. Scores for each E-Risk family were then created by averaging the summary scores of respondents within that family’s neighbourhood. The resulting variable approached normal distribution across the full potential range (*M* = 0.49, *SD* = 0.34, range = 0–1.93). We indexed high levels of neighbourhood disorder as those participants with above average neighbourhood disorder scores (coded 1: 42.2% of participants, *N* = 908).

#### Mental health problems at age 18

At 18 years of age, participants were privately interviewed about past-year symptoms of mental disorder. Ten disorder diagnoses were organised into three domains (externalising, internalising, and thought disorders) based on a reliable latent factor structure for psychopathology previously identified within the E-Risk Study [9]. Full information on individual diagnoses is available in Supplementary Material. Participants were classified as having a research diagnosis of ‘externalising disorder’ when they met diagnostic criteria for ADHD, conduct disorder, alcohol dependence, cannabis dependence, or tobacco dependence. Participants were classified as having a research diagnosis of ‘internalising disorder’ when they met diagnostic criteria for general anxiety disorder, major depressive disorder, post-traumatic stress disorder, or presented at least 2 of 5 eating disorder symptoms from an established screening tool indicating a possible case of anorexia nervosa or bulimia nervosa [50]. Finally, a ‘thought disorder’ classification was based on the definite presence of at least one of seven psychotic symptoms, centred on delusions and hallucinations. A total of 280 E-Risk participants met criteria for more than one classification. Thus, from these three domain-specific classifications, an overall binary outcome for ‘any psychiatric disorder’ was created, denoting the presence of any externalising, internalising, or thought disorder (coded 1), or the absence of all three (0). In the E-Risk study, 47.5% (*N* = 973) of participants met diagnostic criteria for any psychiatric disorder at age 18. Specifically, 31.9% (*N* = 656) met criteria for externalising disorder, 28.5% (*N* = 585) for internalising disorder, and 2.9% (*N* = 59) for thought disorder.

#### Maternal warmth during childhood

Maternal warmth during childhood was assessed using procedures adapted from the Five-Minute Speech Sample method [51, 52]. When children were aged 5 and 10, mothers were asked to speak for 5 minutes about each of the children separately. The speech samples were audiotaped and coded by two independent raters, who had good inter-rater reliability (*r* = 0.90). The warmth expressed by the mother in their interview about the child was coded on a six-point scale from no warmth (0; complete absence of warmth) to high warmth (5; definite warmth, enthusiasm, interest in, and enjoyment of the child). To capture high levels of maternal warmth during childhood, a binary variable was created that indexed the presence of high maternal warmth (i.e., a score of 4–5) at age 5 and/or age 10 (coded 1: 71.4% of participants, *N* = 1466).

#### Sibling warmth during childhood

Sibling warmth during childhood was assessed by asking mothers a series of questions about the quality of their twins’ relationship with one another when the children were aged 7 and 10 [53]. Mothers responded on a three-point scale ranging from 0 ‘no’ to 2 ‘yes’ to six questions (e.g., “do your twins love each other”, “do both your twins do nice things for each other”). Internal consistency at age 7 was α = 0.77 and at age 10 was α = 0.80. As age-7 and age-10 scores were highly correlated (*r* = 0.57, *p* < 0.001), these were summed to create a single composite score (*M* = 19.92, *SD* = 3.35).

#### Perceived social support

Perceived Social Support was assessed at age 18 via self-reports using the multidimensional scale of perceived social support (MSPSS), which assesses individuals' access to supportive relationships with family, friends, and significant others [54]. The 12 items comprise statements such as “There is a special person who is around when I am in need” and “I can count on my friends when things go wrong”. Participants rated these statements as “not true” (0), “somewhat true” (1), or “very true” (2). We summed scores to produce an overall social support scale with higher scores reflecting greater social support (*M* = 20.71, *SD* = 4.35). In addition, the family and friend sub-scales were utilised separately to examine whether social support from either family (*M* = 6.98, *SD* = 1.78) or friends (*M* = 6.71, *SD* = 2.01) was specifically protective.

#### Intelligence quotient (IQ)

Intelligence Quotient (IQ) was tested at age 12 using a short version of the Wechsler Intelligence Scale for Children-Revised (WISC-R) [55] which comprised three subtests (Matrix Reasoning, Information, and Digit Span). We converted the scores into an IQ score according to Sattler [56] and then standardised to a mean of 100 and standard deviation of 15.

#### Family socio-economic status (SES)

Family socio-economic status (SES) was measured at age 5 using a standardised composite of parental income (i.e., total household income), education (i.e., highest parent qualification), and occupation (i.e., highest parent occupation). These three SES indicators were highly correlated (*r* = 0.57–0.67) and loaded significantly onto one latent factor [57]. The population-wide distribution of this latent factor was then divided into tertiles (i.e., low-, medium-, and high-SES).

#### Individual- and family-level covariates

Individual-level covariates included biological sex and participants’ history of emotional and behavioural problems during childhood, including attention-deficit hyperactivity disorder (ADHD) diagnosis, conduct disorder diagnosis, symptoms of depression and anxiety, self-harm and suicide attempts, and psychotic symptoms. Family-level covariates included family SES and family history of psychopathology. Measurement details for all covariates are provided in Supplementary Material.

### Statistical analyses

Analyses were conducted using Stata 15. We accounted for the non-independence of our twin observations in all analyses using the Huber–White variance estimator [58]. Analyses proceeded in three steps. First, we used a series of logistic regression models to examine the separate associations of (i) personal experience of severe physical violence and (ii) high levels of neighbourhood disorder during adolescence with (i) any psychiatric disorder, and then (ii) externalising disorder, (iii) internalising disorder, and (iv) thought disorder at age 18. We also conducted two sensitivity analyses: we examined the association of different types of severe physical violence (i.e., crime victimisation, maltreatment, sexual victimisation, and family violence) with the four age-18 mental health outcomes. We also tested associations using neighbourhood disorder categorised at different thresholds—above the median, above the 75th percentile, and using the full-scale (continuous) measure of neighbourhood disorder.

Second, to investigate the potential cumulative and interactive effects of personal experience of severe physical violence and high neighbourhood disorder, we created a 4-level categorical variable to reflect the four possible combinations of exposure: no exposure (coded 0); personal severe physical violence only (1); high neighbourhood disorder only (2); both personal severe physical violence and high neighbourhood disorder (3). We used Interaction Contrast Ratios (ICRs) to investigate whether personal experience of severe physical violence and high neighbourhood disorder during adolescence combined synergistically to increase the odds of psychiatric disorder at age 18 (indicated by a departure from additivity [59, 60]). This approach uses odds ratios (OR) derived from logistic regression models to estimate the relative excess risk as a result of synergy (i.e., ICR = OR_exposure to both_–OR_personal severe physical violence only_–OR_high neighbourhood disorder only_ + 1).

Third, we used logistic regression to examine whether supportive relationships (maternal warmth, sibling warmth, and perceived social support including family and friend sub-scales), higher IQ, or higher family SES were associated with reduced odds of psychiatric disorder within those violence exposure groups found to be at risk in step 2. We also tested statistical interactions between significant protective factors and adolescent violence exposure in the whole E-Risk sample using logistic regression.

To test the robustness of the associations, all models were adjusted for sex, family history of psychopathology, and childhood emotional and behavioural problems. Family SES was also included as a covariate in models in step 1 and 2; however, because we investigated family SES as a potential protective factor, models in step 3 were not adjusted for this. Because we tested four mental health outcomes in steps 1 and 2, and seven potential protective factors in step 3, we controlled the false discovery rate (FDR) by applying the Benjamini–Hochberg method [61] to each collection of statistical tests. All *p* values are presented in their uncorrected form with an asterisk indicating that the *p* value remained significant after FDR correction. Missing data, which was minimal, were predominantly due to participants missing mental health and personal experience of physical violence data due to not participating in the E-Risk Study follow-up at age 18 (see “Sample” and “Measures” descriptions). This was not associated with living in areas with high levels of neighbourhood disorder (OR = 0.99, *p* = 0.967, 95% CI = 0.63–1.55) and we therefore analysed complete cases.

## Results

### Is personal experience of severe physical violence during adolescence associated with mental health problems at age 18?

Table 1 shows the associations between personal experience of severe physical violence during adolescence and mental health problems at age 18. Having personal experience of severe physical violence was associated with significantly elevated odds of meeting criteria for any psychiatric disorder including externalising, internalising, and thought disorders at age 18. These odds remained significantly elevated after adjusting for covariates. Examination of different types of personal severe physical violence (i.e., crime victimisation, maltreatment, sexual victimisation, and family violence) also showed elevated odds for any psychiatric disorder, externalising disorder, internalising disorder, and thought disorder (see Supplementary Table S2; note these are similar to findings using dimensional measures of mental health in the E-Risk Study; see Schaefer et al. [8]).

**Table 1:** Association of adolescent personal experience of severe physical violence with psychiatric disorders at age 18

Model	Any psychiatric disorder				Externalising disorder				Internalising disorder				Thought disorder			
	*N*	OR	95% CI	*p*	*N*	OR	95% CI	*p*	*N*	OR	95% CI	*p*	*N*	OR	95% CI	*p*
Unadj	2050	4.32	3.43–5.43	< 0.001*	2054	4.43	3.54–5.55	< 0.001*	2050	3.05	2.44–3.80	< 0.001*	2063	3.60	2.17–6.00	< 0.001*
Adj	1973	3.62	2.84–4.62	< 0.001*	1977	3.51	2.76–4.47	< 0.001*	1972	3.13	2.46–3.98	< 0.001*	1987	3.06	1.72–5.42	< 0.001*

CI = confidence interval; OR = Odds ratio. Unadj. = unadjusted associations of violence exposure and age-18 mental health. Adj. = associations adjusted simultaneously for biological sex, family socio-economic status, family history of psychopathology, and childhood emotional and behavioural problems (attention-deficit hyperactivity disorder, conduct disorder, symptoms of depression and anxiety, self-harm and suicide attempts, and psychotic symptoms)

*p values marked by an asterisk remained significant after correction for false discovery rate (FDR) using Benjamini–Hochberg procedure. All models account for the non-independence of twin observations. The sample sizes vary slightly according to the mental health outcome and due to small numbers of participants missing some data on covariates

### Is living in a neighbourhood with high levels of disorder during adolescence associated with mental health problems at age 18?

Table 2 shows the associations between high (i.e., above mean) levels of neighbourhood disorder and mental health problems at age 18. Living in neighbourhoods with high levels of disorder during adolescence was associated with significantly elevated odds of meeting criteria for any psychiatric disorder at age 18. This association held after adjusting for covariates. The elevated odds of externalising, internalising, and thought disorders were no longer statistically significant after adjusting for covariates; however, the effect sizes for internalising and thought disorders were not attenuated. Sensitivity analyses using neighbourhood disorder as (i) a continuous variable or dichotomised at the (ii) median and (iii) 75^th^ percentile revealed a similar pattern of associations (see Supplementary Table S3).

**Table 2:** Association of high levels of neighbourhood disorder during adolescence with psychiatric disorders at age 18

Model	Any psychiatric disorder				Externalising disorder				Internalising disorder				Thought disorder			
	*N*	OR	95% CI	*p*	*N*	OR	95% CI	*p*	*N*	OR	95% CI	*p*	*N*	OR	95% CI	*p*
Unadj	1980	1.59	1.30–1.95	< 0.001*	1984	1.42	1.14–1.76	0.002*	1979	1.32	1.06–1.64	0.014*	1991	1.92	1.10–3.33	0.021*
Adj	1905	1.28	1.02–1.60	0.031*	1909	1.04	0.81–1.33	0.779	1903	1.20	0.95–1.53	0.133	1917	1.86	0.98–3.53	0.059

CI, confidence interval; OR, odds ratio; Unadj., unadjusted associations of violence exposure and age-18 mental health; Adj., associations adjusted simultaneously for biological sex, family socio-economic status, family history of psychopathology, and childhood emotional and behavioural problems (attention-deficit hyperactivity disorder, conduct disorder, symptoms of depression and anxiety, self-harm and suicide attempts, and psychotic symptoms)

*p values marked by an asterisk remained significant after correction for false discovery rate (FDR) using the Benjamini–Hochberg procedure. All models account for the non-independence of twin observations. The sample sizes vary slightly according to the mental health outcome and due to small numbers of participants missing some data on covariates

### Is there a cumulative effect of having both personal experience of severe physical violence and living in a neighbourhood with high levels of disorder during adolescence on mental health at age 18?

**Fig. 1:**
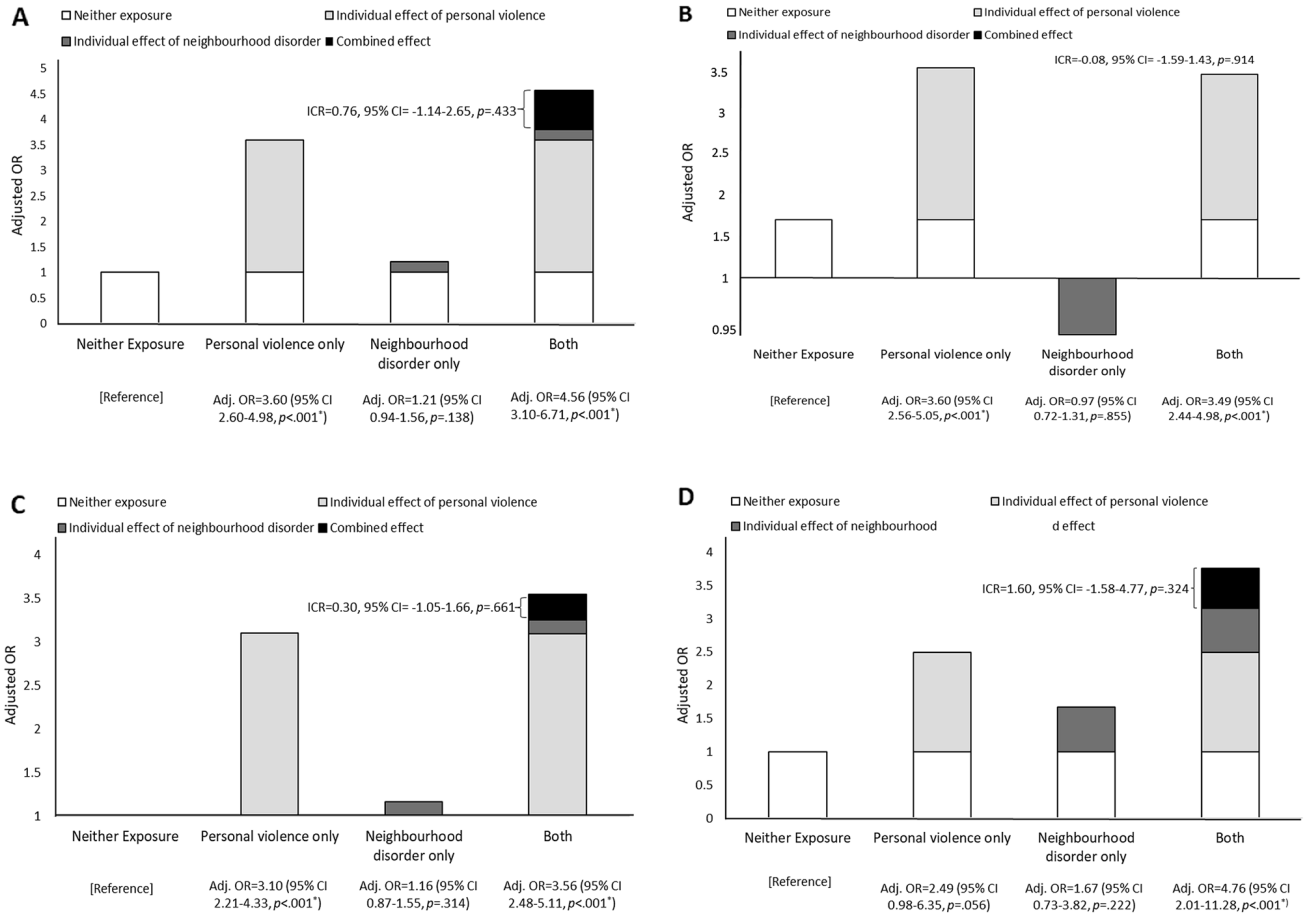
Individual and cumulative effects of personal experience of severe physical violence and high levels of neighbourhood disorder in adolescence on any psychiatric disorder (panel **A**), externalising disorder (panel **B**), internalising disorder (panel **C**), and thought disorder (panel **D**) at age 18. Adj, adjusted; CI, confidence interval; ICR, interaction contrast ratio; OR, odds ratio. Odds ratios are adjusted for biological sex, family socio-economic status, family history of psychopathology, and childhood emotional and behavioural problems (attention-deficit hyperactivity disorder, conduct disorder, symptoms of depression and anxiety, self-harm and suicide attempts, and psychotic symptoms) and the non-independence of twin observations. *p values marked by an asterisk remained significant after correction for the false discovery rate (FDR) using the Benjamini–Hochberg procedure

Of the 502 E-Risk participants with personal experience of severe physical violence in adolescence, half (51.4%, *N* = 258) also lived in neighbourhoods with high levels of disorder. Table 3 shows the prevalence of age-18 psychiatric disorder according to adolescents’ exposure to personal severe physical violence and/or neighbourhood disorder. When both personal experience of severe physical violence and high levels of neighbourhood disorder were considered together, there was evidence that those exposed to both had the highest odds of meeting criteria for any psychiatric disorder at age 18 (Fig. 1, panel A). A similar pattern was evident for internalising and thought disorders—these outcomes were associated most strongly with exposure to both personal experience of severe physical violence and high neighbourhood disorder (Fig. 1, panels C, D). The higher odds were particularly notable for thought disorder. In contrast, the odds of externalising disorder were comparable for those adolescents with only personal experience of severe physical violence and those who also lived in high disorder neighbourhoods (Fig. 1, panel B). Adolescents who lived in neighbourhoods with high levels of disorder but did not have personal experience of severe physical violence were no more likely to meet criteria for a psychiatric disorder at age 18 than the non-exposed group.

**Table 3:** Prevalence of age-18 psychiatric disorders according to adolescent violence exposure

Adolescent violence exposure	*N*	Any psychiatric disorder present *n* (%)	Externalising disorder present *n* (%)	Internalising disorder present *n* (%)	Thought disorder present *n* (%)
None^a^	923	325 (35.5)	201 (21.9)	194 (21.1)	14 (1.5)
Personal severe physical violence only	230	160 (69.6)	126 (55.0)	104 (45.2)	10 (4.3)
Neighbourhood disorder only	583	252 (43.6)	147 (25.3)	142 (24.6)	14 (2.4)
Both^b^	258	198 (77.0)	156 (60.7)	120 (47.4)	19 (7.4)

^a^Includes adolescents with no exposure to either personal severe physical violence or high neighbourhood disorder during adolescence

^b^Includes adolescents with exposure to both personal severe physical violence and high neighbourhood disorder

Interaction contrast ratios for all mental health outcomes were non-significant showing that the combined effect of exposure to personal severe physical violence and high neighbourhood disorder was not significantly different to their summed effect.

### Do supportive relationships, higher IQ, or higher family SES protect against mental health problems for those violence-exposed groups who are at risk?

Having established that adolescents’ personal experience of severe physical violence—with or without high levels of neighbourhood disorder—is associated with elevated odds of mental health problems across externalising, internalising, and thought disorders, we focus here just on the overarching ‘any psychiatric disorder’ outcome. Despite their elevated risk, in the E-Risk sample, just over 30% (*N* = 70) of adolescents who personally experienced only severe physical violence and 23% (*N* = 59) of those exposed to both severe physical violence and high neighbourhood disorder did not meet diagnostic criteria for a psychiatric disorder at age 18. We therefore examined whether supportive relationships, higher IQ, and higher family SES were operating as protective factors in these two violence-exposed subsamples (see Supplementary Tables S4 and S5 for descriptive statistics).

The results (Table 4) show that having family support at age 18 was associated with lower odds of any psychiatric disorder in adolescents exposed only to severe physical violence and those who also lived with high levels of neighbourhood disorder (though the latter did not remain significant after correction for FDR). For those with personal experience of only severe physical violence, higher overall levels of perceived social support and support from friends specifically were also associated with lower odds of any psychiatric disorder. Interestingly, the results showed that higher IQ, sibling and maternal warmth during childhood, and family SES were not significantly protective against age-18 psychiatric disorder in the violence-exposed subsamples.

**Table 4:** Association between potential protective factors and any psychiatric disorder at age 18 among adolescents exposed to (i) personal severe physical violence only and (ii) both personal severe physical violence and high neighbourhood disorder

Potential protective factors	Personal severe physical violence only			Both personal severe physical violence and high neighbourhood disorder		
	Adjusted OR^a^	95% CI	*p*	Adjusted OR^a^	95% CI	*p*
*Maternal warmth during childhood*						
Low	[Reference]			[Reference]		
High	0.65	0.31–1.35	0.248	0.79	0.39–1.61	0.522
*Sibling warmth during childhood*	0.97	0.88–1.07	0.571	0.97	0.86–1.09	0.578
*Perceived social support at age 18*	0.91	0.85–0.98	0.017*	0.95	0.87–1.03	0.211
*Family support subscale at age 18*	0.76	0.62–0.94	0.010*	0.82	0.70–0.98	0.026
*Friend support subscale at age 18*	0.80	0.68–0.94	0.005*	0.97	0.83–1.14	0.749
*IQ at age 12*	0.99	0.96–1.01	0.172	1.01	0.98–1.03	0.668
*Family SES at age 5*						
Low	[Reference]			[Reference]		
Mid	2.21	0.97–5.04	0.059	0.90	0.43–1.90	0.785
High	0.86	0.39–1.90	0.709	1.12	0.37–3.40	0.846

CI, confidence interval; IQ, intelligence quotient; *OR*, odds ratio; *SES*, socio-economic status

^a^Adjusted simultaneously for biological sex, family history of psychopathology, and childhood emotional and behavioural problems (attention-deficit hyperactivity disorder, conduct disorder, symptoms of depression and anxiety, self-harm and suicide attempts, and psychotic symptoms).

*p values marked by an asterisk remained significant after correction for the false discovery rate (FDR) using the Benjamini–Hochberg procedure. All models account for the non-independence of twin observations

Next, we tested for interactions between perceived social support, including family and friend sub-scales and adolescent violence exposure. None of these interactions were statistically significant (all *p*’s > 0.05, see Supplementary Table S6).

## Discussion

This study examined the association of adolescent violence exposure at the inter-personal and community level with mental health at the transition to adulthood. We found elevated odds for meeting diagnostic criteria for any psychiatric disorder (including externalising, internalising, and thought disorders) for adolescents with personal experience of severe physical violence. We also found evidence of a cumulative association with internalising and thought disorders for adolescents with personal experience of severe physical violence who also lived in neighbourhoods with high levels of disorder. Higher levels of perceived support (including from family and friends) at age 18 were associated with a reduced likelihood of psychiatric disorder following personal experiences of severe physical violence whereas only perceived support from family was related to reduced odds for those who were additionally exposed to neighbourhood disorder (though this association was not statistically significant). These results hint at a protective effect; however, perhaps, due to a lack of statistical power, interactions with violence exposure were not statistically significant.

Our finding that adolescents who personally experienced violence and lived in neighbourhoods with high levels of disorder had the greatest odds of internalising and thought disorders is consistent with the cumulative stress hypothesis and existing research on poly-victimisation [9]. Interestingly, there was no cumulative association with externalising disorder—living in an area with high neighbourhood disorder during adolescence did not contribute any additional risk compared to having only personal experience of violence. This is in line with previous findings by Meltzer et al. [62] that adolescents’ feeling of safety in their neighbourhood was related to emotional disorders but not conduct disorder, suggesting that neighbourhood disorder may be differentially associated with internalising and externalising problems. We speculate that living in dangerous or threatening communities may promote maladaptive cognitive styles such as biased threat perception and paranoia that are implicated in disorders such as anxiety and psychosis [63, 64]. However, future studies that examine potential mechanisms and qualitative studies to better understand individuals’ experiences are needed to investigate this further.

We also revealed a stronger association between personal experiences of severe physical violence and mental health (compared to neighbourhood disorder). This may be understood in terms of the proximity of the violence to the adolescent; violence at the inter-personal level is a more proximal exposure than violence that occurs at the community level. Therefore, those who live in a neighbourhood with high levels of disorder may be able to ignore or more easily distance themselves from this whereas having personal experience of violence is likely very distressing and difficult to get respite from, especially if it is ongoing. Relatedly, because we used reports of neighbourhood disorder from near-by residents rather than the participants themselves, adolescents may not have perceived their neighbourhood in the same way [19]. While our approach has the methodological advantage of avoiding same-source bias, which may inflate associations with mental health [65], a link between the two may depend on adolescents’ themselves perceiving there to be a high level of neighbourhood disorder where they live. Indeed, studies that have utilised both perceptions of violence in the neighbourhood and officially reported crime statistics suggest that it is people’s perceptions of their neighbourhood that are most relevant for their mental health [15, 18, 19].

Consistent with a wealth of existing research demonstrating the benefits of social support for health and wellbeing [66–69], we found evidence that perceived social support at age 18 helped reduce the likelihood that violence-exposed adolescents meet criteria for psychiatric disorder at the transition to adulthood. This suggests that having someone that adolescents can share their experiences and worries with and seek emotional support and advice from is important for maintaining good mental health. Given the prominence of peer friendships and individuals’ increasing independence from their family during adolescence, it is notable that perceived support from family also helped maintain mental health following violence exposure. In fact, for those exposed to both personal violence and neighbourhood disorder, it was higher levels of family support (not friend support) at age 18 that was associated (albeit non-significantly after FDR correction) with a reduced likelihood of psychiatric disorder. On the contrary, maternal warmth and sibling warmth assessed during childhood were not associated with a reduced likelihood of meeting criteria for psychiatric disorder. This may be because the level of sibling and maternal warmth is too low among those children who go on to experience violence in adolescence, or it may be that it is adolescents’ perception of supportive relationships that are currently available to them that is most valuable for maintaining mental health in the face of violence exposure.

### Strengths and limitations

Study strengths include the use of a large nationally representative sample, longitudinal study design with excellent participant retention, and inclusion of a broad range of covariates to limit alternative interpretations. Nonetheless, we also acknowledge several limitations. First, participants’ mental health problems and personal experience of violence were both self-reported at age 18. Although adolescents are likely to be the most knowledgeable about their experiences, their current mental health may have impacted their reporting of violence exposure, resulting in reverse causation. For example, depressive disorder may bias recall of negative experiences [70] or improve the accuracy of reporting (so-called ‘depressive realism’ [71]). Cognitive avoidance strategies can also affect the retrieval of memory in individuals with post-traumatic stress [72]. However, the prevalence of violent experiences during adolescence in the E-Risk study is comparable to other UK studies, suggesting that these were not significantly under- or over-reported [24]. Similarly, perceived social support was reported at age 18 which has implications for interpreting its association with age-18 mental health. We did control for a range of earlier emotional and behavioural problems to try to rule out reverse causation, but it remains possible that the severity of adolescents’ mental health symptoms during the past year impacted their perceived level of support. Second, neighbourhood disorder was measured only once during adolescence when participants were approximately 13 years old. The majority of participants (71.4%, *N* = 1475) remained living at the same home address between the ages of 12 and 18, though levels of disorder within their neighbourhood may have changed over time. Third, our measure of neighbourhood disorder considers the immediate environment where E-Risk participants live. However, adolescents likely also spend time in other neighbourhoods (e.g., for education, work, and leisure) which may expose them to high levels of disorder that could impact their risk for later mental health problems. Fourth, items measuring neighbourhood disorder were averaged to create a total score. This approach considers less severe forms of disorder (such as graffiti) as being equal to more severe, potentially less common, forms (e.g., assault) which may underestimate the level of disorder in a neighbourhood. There are alternative ways of aggregating items to account for their differences in severity [73]. Fifth, we focus on adolescents’ personal experience of severe physical violence; therefore, our findings may not generalise to other harmful experiences, such as non-physical bullying, cyber-bullying, and emotional abuse. Our findings also do not inform about associations with mental health beyond the age of 18; some of those who did not report symptoms may go on to develop psychiatric disorders in the future. Sixth, although we focused on disorders, there are other ways of conceptualising psychopathology (e.g., symptom continuum [74]) which our results do not necessarily inform about. Finally, our findings were based on a sample of twins, and these may differ from non-twins. However, the E-Risk sample is representative of UK families in terms of socio-economic distribution [75] and neighbourhood deprivation [76] and the prevalence of violence victimisation and mental health problems has been shown to be comparable between twins and non-twins [77].

## Conclusion

Our findings reaffirm the need for early intervention to support adolescents who experience violence and highlights the vulnerability of those whose personal experience takes place in the context of community-level violence. Interventions focused on improving perceived social support by increasing the availability of supportive people or boosting adolescents’ perceptions of their existing support network may be effective in protecting their mental health.

## Supplementary Information

Below is the link to the electronic supplementary material.

Supplementary file1 (DOCX 80 KB)

## Data Availability

The dataset reported in the current article is not publicly available due to lack of informed consent and ethical approval for open access, but is available on request by qualified scientists. Requests require a concept paper describing the purpose of data access, ethical approval at the applicant’s institution, and provision for secure data access (for further details, see here: https://sites.duke.edu/moffittcaspiprojects/concept-paper-template/). We offer secure access on the Duke University and King's College London campuses. All data analysis scripts and results files are available for review on request from the corresponding author.
