# Supplementary material for: Violent experiences and neighbourhoods during adolescence: understanding and mitigating the association with mental health at the transition to adulthood in a longitudinal cohort study
Source: Soc Psychiatry Psychiatr Epidemiol. 2022 Aug 9;57(12):2379–91. doi: 10.1007/s00127-022-02343-6 (PMC9672016; doi:10.1007/s00127-022-02343-6)
Supplement: Supplementary file 1 — Supplementary file1 (DOCX 80 KB) [file 127_2022_2343_MOESM1_ESM.docx]

**Supplementary Material**

For Latham et al. *“Violent experiences and neighbourhoods during adolescence: understanding and mitigating the association with mental health at the transition to adulthood in a longitudinal cohort study”*

**Methods**

**Study Cohort**

Participants were members of the Environmental Risk (E-Risk) Longitudinal Twin Study, which tracks the development of a nationally representative birth cohort of 2,232 British twin children. The sample was drawn from a larger birth register of twins born in England and Wales in 1994-1995 [1]. Full details about the sample are reported elsewhere [2]. Briefly, the E-Risk sample was constructed in 1999-2000, when 1,116 families (93% of those eligible) with same-sex 5-year-old twins participated in home-visit assessments. This sample comprised 56% monozygotic (MZ) and 44% dizygotic (DZ) twin pairs; sex was evenly distributed within zygosity (49% male). Families were recruited to represent the UK population of families with newborns in the 1990s, on the basis of residential location throughout England and Wales and mother’s age. Teenaged mothers with twins were over-selected to replace high-risk families who were selectively lost to the register through non-response. Older mothers having twins via assisted reproduction were under-selected to avoid an excess of well-educated older mothers.

Follow-up home-visits were conducted when children were aged 7, 10, 12 and 18 (participation rates were 98%, 96%, 96% and 93%, respectively). Home-visits at ages 5, 7, 10, and 12 years included assessments with participants as well as their mother (or primary caregiver); the home-visit at age 18 included interviews only with the participants. Each participant in a twin pair was assessed by a different interviewer. There were 2,066 E-Risk participants who were assessed at age 18. The average age of the participants at the time of the assessment was 18.4 years (SD = 0.36); all interviews were conducted after the 18th birthday. There were no differences between those who did and did not take part at age 18 in terms of socioeconomic status (SES) assessed when the cohort was initially defined (χ^2^ = 0.86, p = 0.65), age-5 IQ scores (t = 0.98, p = 0.33), age-5 internalising or externalising behaviour problems (t = 0.40, p = 0.69 and t = 0.41, p = 0.68, respectively), or childhood poly-victimisation (z=0.51, p=0.61). The study sample represents the full range of socioeconomic conditions in Great Britain, as reflected in the families’ distribution on a neighbourhood-level socioeconomic index (ACORN [A Classification of Residential Neighbourhoods] developed by CACI Inc. for commercial use [3]. E-Risk families ACORN distribution closely matches that of households nation-wide: 25.6% of E-Risk families live in “wealthy achiever” neighbourhoods compared to 25.3% of households nation-wide; 5.3% vs 11.6% live in “urban prosperity” neighbourhoods; 29.6% vs 26.9% live in “comfortably off” neighbourhoods; 13.4% vs 13.9% live in “moderate means” neighbourhoods; and 26.1% vs 20.7% live in “hard-pressed” neighbourhoods. E-Risk underrepresents urban prosperity neighbourhoods because such households are likely to be childless.

Parents gave informed consent and twins gave assent between 5-12 years and then informed consent at age 18. The Joint South London and Maudsley and the Institute of Psychiatry Research Ethics Committee approved each phase of the study.

***Supplementary Figure S1.*** The E-Risk Study families’ addresses are a near-perfect match to the deciles of the UK government’s Index of Multiple Deprivation.

*Note.* This histogram shows E-Risk families’ addresses are a near-perfect match to the deciles of the UK’s 2015 Lower-layer Super Output Area (LSOA) Index of Multiple Deprivation (IMD) which averages 1,500 residents (or 650 households each); approximately 10% (dotted red line) of the E-Risk cohort fills each of the IMD’s 10% bands, indicating that the E-Risk cohort accurately represents the distribution of deprivation in the UK.

**Measures**

**Personal experience of severe physical violence during adolescence.** At age 18, participants were interviewed face-to-face about exposure to a range of adverse experiences between 12 and 18 years using the Juvenile Victimisation Questionnaire (JVQ) [4, 5] adapted as a clinical interview [6]. Our adapted JVQ comprised 45 questions covering different forms of victimisation grouped into seven categories: crime victimisation, peer/sibling victimisation, internet/mobile phone victimisation, sexual victimisation, family violence, maltreatment, and neglect (see Fisher et al. [6] for full details). Within each pair of twins in our cohort, cotwins were interviewed separately by different interviewers and were assured of the confidentiality of their responses. The participants were advised that confidentiality would only be broken if they told the interviewers that they were in immediate danger of being hurt, and in such situations, the project leader would be informed and would contact the participant to discuss a plan for safety.

Each JVQ question was asked for the period “since you were 12”. Age 12 is a salient age for our participants because it is the age when British children leave primary school to enter secondary school. Participants were given the option to say “yes” or “no” as to whether each type of victimisation had occurred in the reporting period. If an experience was endorsed within a victimisation category, follow-up questions were asked concerning how old the participant was when it (first) happened, whether the participant was physically injured in the event, whether the participant was upset or distressed by the event, and how long it went on for (by marking the number of years on a Life History Calendar; Caspi et al. [7]). In addition, the interviewer wrote detailed notes based on the participant’s description of the worst event. If multiple experiences were endorsed within a victimisation category, the participant was asked to identify and report about his or her worst experience.

All information from the JVQ interview was compiled into victimisation dossiers. Using these dossiers, an expert in victimology (Dr Helen Fisher) and 3 other members of the E-Risk team evaluated whether each participant was exposed to any physical violence, whether in the family, by peers, or by people in the wider environment, based on the entire dossier of victimisation experiences. This “any physical violence” exposure variable was rated on a 6-point scale: 0 = not exposed, then 1–5 for increasing levels of severity, with severity linked to frequency of occurrence of non-injurious physical attacks at the lower end of the scale (1–3) and the likelihood of incurring an injury and the seriousness of this injury indicating severity at the upper end of the scale (4–5) (see Table S1 for coding detail). This rating included violence directed toward the participants themselves as well as violence they witnessed between other people. Each twin’s dossier was evaluated separately, and we did not use information provided in the cotwin’s dossier about his or her own or shared victimisation experiences to rate direct or witnessed violence exposure for the target twin. To index the most severe experiences of violence we dichotomised this variable such that those scoring at the upper end of the severity scale (4-5) were identified as having personal experience of severe violence (coded 1: 24.3% of participants, N=502). For sensitivity analyses using specific types of physical violence, measures of crime victimisation, maltreatment, sexual victimisation and family violence were dichotomised as scores of 0-3 versus 4-5 to identify those at the upper end of the severity scale (coded 1). In the E-Risk sample, 19.3% (N=398) were exposed to severe crime victimisation, 3.3% (N=67) to severe maltreatment, 2.6% (N=53) to severe sexual victimisation and 12.1% (N=250) to severe family violence during adolescence.

**Table S1.** Anchor Points for Rating Adolescent Physical Violence Exposure

| **Any physical violence exposure** | |
| --- | --- |
| **5** | Life threats or severe injury likely to twin or witnessed to others |
| **4** | Injury likely but no threat to life to twin or witnessed to others |
| **3** | Repeated non-injurious physical attacks to twin or witnessed to others |
| **2** | Occasional non-injurious physical attacks to twin or witnessed to others |
| **1** | Single non-injurious physical attack to twin or witnessed to others |
| **0** | No physical attacks to twin or witnessed to others |

**Mental health problems at age 18.**

***Externalising disorders.*** Past-year symptoms of five externalising disorders were assessed as part of private interviews with participants at age 18. Attention-deficit/hyperactivity disorder (ADHD) diagnosis was based on *DSM-5* criteria [8]. Diagnoses of moderate conduct disorder (CD), alcohol dependence, and cannabis dependence were made using *DSM-IV* criteria [9]. Tobacco dependence was diagnosed using the Fagerström Test for Nicotine Dependence [10]. Participants meeting criteria for one or more of these diagnoses were classified as having ‘externalising disorder’.

***Internalising disorders*.** Past-year symptoms of four internalising disorders were assessed during private interviews at the age-18 follow-up. Generalised anxiety disorder (GAD) and major depressive disorder (MDD) diagnoses were based on *DSM-IV* criteria [9]. Post-traumatic stress disorder (PTSD) diagnosis was derived using *DSM-5* criteria [8]. Eating disorder diagnosis was based on a cut-off of two or more symptoms on the five-item SCOFF screening tool, which consistently detects all positive cases of anorexia nervosa and bulimia nervosa in patient samples [11, 12]. Participants who met criteria for one or more of these four diagnoses were classified as having ‘internalising disorder’.

***Thought disorder*.** Occurrence of psychotic symptoms since age 12 was assessed at age 18. This interview procedure, which is the same as that used at age 12 follow up, has been described in detail elsewhere [13]. Seven items pertaining to delusions and hallucinations were assessed, including “Have other people read your thoughts?”, “Have you thought you were being followed or spied on?”, and “Have you heard voices that other people cannot hear?”. Interviewers coded each item as ‘not’, ‘probably’, or ‘definitely’ present, with coding subsequently verified by a senior psychiatrist. Participants who experienced at least one definite psychotic symptom were classified as having thought disorder. This measure has good construct validity, as it was shown to share many of the genetic, social, neurodevelopmental, and behavioural risk factors and correlates as adult schizophrenia [13].

***Any psychiatric disorder*.** Based on the binary classifications generated for each of the three psychopathological domains, an overall binary outcome for ‘any psychiatric disorder’ was created, where a score of 1 denoted the presence of any internalising, externalising, or thought disorder, and a score of 0 indicated that all three were absent.

**Individual- and family-level covariates.**

***Sex*** was reported by mothers at study baseline (1 = male; 2 = female).

***Attention deficit hyperactivity disorder (ADHD) diagnosis.*** We ascertained ADHD diagnosis on the basis of mother and teacher reports of 18 symptoms of inattention and hyperactivity-impulsivity according to DSM-IV criteria [9]. Participants had to have six or more symptoms reported by mothers or teachers in the past 6 months, and the other informant must have endorsed at least two symptoms. For the current study, we considered participants to have a diagnosis of childhood ADHD if they met criteria at age 5, 7, 10 or 12. Participants who had ADHD information on at least two of the four childhood assessments and did not meet diagnostic criteria at any available assessments were classified as not having the disorder in childhood. Two children whose symptoms fell below threshold for diagnosis but were taking ADHD medication were included in the ADHD group. In total, 266 participants (12.1%) met criteria for ADHD in childhood.

***Conduct disorder diagnosis.*** We derived a diagnosis of children’s conduct disorder on the basis of mothers’ and teachers’ reports on 14 of 15 items from DSM-IV [9] criteria for conduct disorder (excluding “forced sexual activity” criteria, given the age of the participants). We considered participants to have a diagnosis of conduct disorder if they met five or more criteria at age 5, 7, 10, or 12. In total, 349 participants (15.8%) met criteria for conduct disorder in childhood.

***Anxiety symptoms*** were assessed when children were aged 12 via private interviews using the 10-item version of the Multidimensional Anxiety Scale for Children (MASC) [14]. Items were summed to indicate severity of anxiety (*M* = 7.62, *SD* = 3.04).

***Depression symptoms*** were assessed when children were aged 12 via private interviews using the 27-item Children’s Depression Inventory (CDI) [15]. Items were summed to indicate severity of depression (*M* = 3.11, *SD* = 5.32).

***Self-harm and suicide attempts*** were captured by asking mothers whether each twin had ever deliberately harmed him/herself or attempted suicide in the previous six months, as part of a face-to-face interview when the children were aged 12. Mothers who responded positively to this question were asked to provide a description of the event(s). An independent rater blind to other data subsequently used the interview notes to verify that the description provided was clearly an act of self-harm [16]. We asked only mothers, and not children, to report the child’s self-harm because of ethical considerations. From this, 2.9% (*n* = 62) of Study members had displayed any self-harming or suicidal behaviour. Examples of self-harming behaviours included cutting and biting arms, pulling out clumps of hair, banging head against walls, and attempted suicides by strangulation.

***Psychotic symptoms*** were evaluated in private interviews by mental health trainees or professionals when children were aged 12. This interview has been described in detail previously [13]. To summarise, each child was privately interviewed about seven psychotic symptoms pertaining to delusions and hallucinations, with items including “have other people ever read your thoughts?”, “have you ever thought you were being followed or spied on?” and “have you ever heard voices that other people cannot hear?”. A conservative approach was taken in designating a child's report as a symptom. First, the interviewer probed responses using standard prompts designed to discriminate between experiences that were plausible (e.g., “I was followed by a man after school”) and potential symptoms (e.g., “I was followed by an angel who guards my spirit”) and wrote down the child's narrative description of the experience. Second, validity of symptoms was verified by a psychiatrist expert in schizophrenia, a psychologist expert in interviewing children, and a child and adolescent psychiatrist. Third, because children were twins, experiences limited to the twin relationship (e.g., “My twin and I often know what each other are thinking”) were coded as ‘not a symptom’. Children were only classified as experiencing psychotic symptoms if they reported at least one definite symptom. At age 12, 5.9% (*n* = 125) of children reported experiencing at least one definite psychotic symptom.

***Family socio-economic status (SES)*** was defined at age 5 using a standardised composite of parental income (i.e., total household income), education (i.e., highest parent qualification), and occupation (i.e., highest parent occupation). These three SES indicators were highly correlated (*r* = 0.57-0.67) and loaded significantly onto one latent factor [17]. The population-wide distribution of this latent factor was then divided into tertiles (i.e., low-, medium-, and high-SES).

***Family history of psychopathology*** was assessed when children were aged 12. In private interviews, the twins’ mothers reported on her own history of DSM disorders, along with that of her biological mother, father, sisters, and brothers, as well as the twins’ biological father [18]. This was converted to a proportion (0–1) of family members with a history of psychiatric disorder (*M* = 0.37, *SD* = 0.27) [19].

**Results**

**Table S2.** Associations Between Different Types of Personal Severe Physical Violence in Adolescence and Psychiatric Disorders at Age 18.

|  |  | **Any psychiatric disorder** | | | | **Externalising disorder** | | | | **Internalising disorder** | | | | **Thought disorder** | | | |
| --- | --- | --- | --- | --- | --- | --- | --- | --- | --- | --- | --- | --- | --- | --- | --- | --- | --- |
|  | Model | N | OR | 95% CI | *p* | N | OR | 95% CI | *p* | N | OR | 95% CI | p | N | OR | 95% CI | *P* |
| Crime victimisation | Unadj. | 2050 | 3.64 | 2.85 – 4.66 | <.001^*^ | 2054 | 3.90 | 3.08 – 4.94 | <.001^*^ | 2050 | 2.78 | 2.19 – 3.52 | <.001^*^ | 2063 | 3.22 | 1.93 – 5.36 | <.001^*^ |
|  | Adj. | 1973 | 3.14 | 2.43-4.07 | <.001^*^ | 1997 | 3.25 | 2.51 - 4.22 | <.001^*^ | 1972 | 2.79 | 2.17 - 3.60 | <.001^*^ | 1987 | 2.61 | 1.45- 4.70 | .001^*^ |
| Maltreatment | Unadj. | 2048 | 6.65 | 3.19 – 13.86 | <.001^*^ | 2052 | 7.31 | 4.01 – 13.32 | <.001^*^ | 2047 | 4.07 | 2.40 – 6.92 | <.001^*^ | 2063 | 7.97 | 4.08 – 15.59 | <.001^*^ |
|  | Adj. | 1973 | 4.19 | 1.88-9.35 | <.001^*^ | 1977 | 5.09 | 2.56 - 10.09 | <.001^*^ | 1972 | 3.41 | 1.74 - 6.69 | <.001^*^ | 1987 | 5.20 | 1.92 - 14.08 | .001^*^ |
| Sexual victimisation | Unadj. | 2044 | 29.69 | 7.17 – 122.82 | <.001^*^ | 2048 | 4.32 | 2.46 – 7.61 | <.001^*^ | 2043 | 9.96 | 5.10 – 19.47 | <.001^*^ | 2059 | 12.20 | 6.25 – 23.81 | <.001^*^ |
|  | Adj. | 1969 | 36.33 | 4.84 - 272.67 | <.001^*^ | 1973 | 3.55 | 1.76 - 7.18 | <.001^*^ | 1968 | 6.28 | 2.77 - 14.26 | <.001^*^ | 1983 | 8.39 | 3.08 - 22.81 | <.001^*^ |
| Family violence | Unadj. | 2047 | 3.37 | 2.50 – 4.56 | <.001^*^ | 2051 | 2.64 | 1.99 – 3.50 | <.001^*^ | 2046 | 2.97 | 2.23 – 3.95 | <.001^*^ | 2062 | 3.07 | 1.74 – 5.44 | <.001^*^ |
|  | Adj. | 1972 | 2.86 | 2.06 - 3.98 | <.001^*^ | 1976 | 2.14 | 1.57 - 2.92 | <.001^*^ | 1971 | 2.83 | 2.07 - 3.86 | <.001^*^ | 1986 | 2.25 | 1.17 - 4.35 | .016^*^ |

*Note*. CI=confidence interval; OR=Odds ratio. Unadj. = unadjusted associations of violence exposure and age-18 mental health. Adj. = associations adjusted simultaneously for biological sex, family socio-economic status, family history of psychopathology, and childhood emotional and behavioural problems (attention deficit and hyperactivity disorder, conduct disorder, symptoms of depression and anxiety, self-harm and suicide attempts, and psychotic symptoms). ^*^*P*-values marked by an asterisk remained significant after correction for the false discovery rate (FDR) using the Benjamini-Hochberg procedure. All models account for the non-independence of twin observations. The sample sizes vary slightly according to the mental health outcome and due to small numbers of participants missing some data on covariates.

**Table S3**. Association of Neighbourhood Disorder with Psychiatric Disorders at Age 18

|  |  | **Any psychiatric disorder** | | | | **Externalising disorder** | | | | **Internalising disorder** | | | | **Thought disorder** | | | |
| --- | --- | --- | --- | --- | --- | --- | --- | --- | --- | --- | --- | --- | --- | --- | --- | --- | --- |
|  | **Model** | **N** | **OR** | **95% CI** | ***P*** | **N** | **OR** | **95% CI** | ***P*** | **N** | **OR** | **95% CI** | **p** | **N** | **OR** | **95% CI** | ***P*** |
| High (above mean) levels of neighbourhood disorder^1^ | Unadj. | 1980 | 1.59 | 1.30–1.95 | <.001^*^ | 1984 | 1.42 | 1.14–1.76 | .002^*^ | 1979 | 1.32 | 1.06–1.64 | .014^*^ | 1991 | 1.92 | 1.10– 3.33 | .021^*^ |
|  | Adj. | 1905 | 1.28 | 1.02–1.60 | .031^*^ | 1909 | 1.04 | 0.81–1.33 | .779 | 1903 | 1.20 | 0.95–1.53 | .133 | 1917 | 1.86 | 0.98– 3.53 | .059 |
| Full scale neighbourhood disorder | Unadj. | 1980 | 1.77 | 1.31–2.39 | <.001^*^ | 1984 | 1.59 | 1.17–2.17 | .003^*^ | 1979 | 1.53 | 1.12–2.09 | .007^*^ | 1991 | 2.35 | 1.20– 4.58 | .012^*^ |
|  | Adj. | 1905 | 1.22 | 0.86–1.71 | .265 | 1909 | 1.00 | 0.69–1.45 | .986 | 1903 | 1.24 | 0.86–1.77 | .245 | 1917 | 2.33 | 1.08– 5.02 | .032 |
| Above median levels of neighbourhood disorder | Unadj. | 1980 | 1.48 | 1.21-1.82 | <.001^*^ | 1984 | 1.43 | 1.15-1.77 | .001^*^ | 1979 | 1.25 | 1.00-1.56 | .048 | 1991 | 2.04 | 1.14-3.64 | .016^*^ |
|  | Adj. | 1905 | 1.18 | 0.94-1.47 | .149 | 1909 | 1.03 | 0.80-1.31 | .829 | 1903 | 1.15 | 0.90-1.47 | .252 | 1917 | 1.87 | 0.96-3.64 | .064 |
| Above 75^th^ centile levels of neighbourhood disorder | Unadj | 1980 | 1.57 | 1.24-1.98 | <.001^*^ | 1984 | 1.43 | 1.12-1.82 | .004^*^ | 1979 | 1.46 | 1.14-1.87 | .003^*^ | 1991 | 1.91 | 1.08-3.38 | .026 |
|  | Adj. | 1905 | 1.19 | 0.92-1.53 | .184 | 1909 | 1.01 | 0.77-1.32 | .968 | 1903 | 1.22 | 0.93-1.60 | .144 | 1917 | 1.79 | 0.97-3.32 | .064 |

*Note*. ^1^Results as reported in the main manuscript for comparative purposes. CI=confidence interval; OR=Odds ratio. Unadj.=unadjusted associations of violence exposure and age-18 mental health. Adj.=associations adjusted simultaneously for biological sex, family socio-economic status, family history of psychopathology, and childhood emotional and behavioural problems (attention deficit and hyperactivity disorder, conduct disorder, symptoms of depression and anxiety, self-harm and suicide attempts, and psychotic symptoms). ^*^*P*-values marked by an asterisk remained significant after correction for the false discovery rate (FDR) using the Benjamini-Hochberg procedure. All models account for the non-independence of twin observations. The sample sizes vary slightly according to the mental health outcome and due to small numbers of participants missing some data on covariates.

**Table S4.** Descriptive Statistics for Potential Protective Factors Among Adolescents Exposed to Personal Severe Physical Violence Only

|  | **Any psychiatric disorder present** | **Any psychiatric disorder absent** |
| --- | --- | --- |
| **Potential protective factors** | **M (SD) or Frequency (%)** | **M (SD) or Frequency (%)** |
| Maternal warmth during childhood |  |  |
| Low | 51 (34.0) | 15 (23.4) |
| High | 99 (66.0) | 49 (76.6) |
| Sibling warmth during childhood | 19.36 (3.41) | 19.87 (3.47) |
| Perceived social support at age 18 | 18.68 (5.40) | 20.69 (4.49) |
| Family support subscale at age 18 | 5.98 (2.42) | 7.11 (1.68) |
| Friend support subscale at age 18 | 5.92 (2.42) | 6.90 (1.69) |
| IQ at age 12 | 96.60 (15.56) | 103.39 (16.03) |
| Family socio-economic status at age 5 |  |  |
| Low | 55 (34.4) | 20 (28.6) |
| Mid | 60 (37.5) | 18 (25.7) |
| High | 45 (28.1) | 32 (45.7) |

*Note*. IQ=intelligence quotient; M=mean; SD=standard deviation.

**Table S5.** Descriptive Statistics for Potential Protective Factors Among Adolescents Exposed to Both Personal Severe Physical Violence and High Neighbourhood Disorder

|  | **Any psychiatric disorder present** | **Any psychiatric disorder absent** |
| --- | --- | --- |
| **Potential protective factors** | **M (SD) or Frequency (%)** | **M (SD) or Frequency (%)** |
| Maternal warmth during childhood |  |  |
| Low | 79 (45.1) | 19 (33.3) |
| High | 96 (54.9) | 38 (66.7) |
| Sibling warmth during childhood | 18.69 (3.50) | 19.69 (3.05) |
| Perceived social support at age 18 | 18.76 (5.36) | 20.39 (5.18) |
| Family support subscale at age 18 | 5.82 (2.54) | 6.83 (2.05) |
| Friend support subscale at age 18 | 6.15 (2.47) | 6.49 (2.27) |
| IQ at age 12 | 92.63 (14.69) | 94.62 (12.54) |
| Family socio-economic status at age 5 |  |  |
| Low | 122 (61.6) | 32 (54.2) |
| Mid | 56 (28.3) | 21 (35.6) |
| High | 20 (10.1) | 6 (10.2) |

*Note*. IQ=intelligence quotient; M=mean; SD=standard deviation.

**Table S6.** Interaction Between Each Protective Factor and Adolescent Violence Exposure in Association with Any Psychiatric Disorder at Age 18.

| **Interaction term** | **Adjusted OR^a^** | **95% CI** | ***P*** |
| --- | --- | --- | --- |
| Perceived social support at 18 * violence exposure |  |  |  |
| None^b^ | [reference] | | |
| Personal severe physical violence only | 0.98 | 0.90 **–** 1.07 | .663 |
| Both^c^ | 1.00 | 0.92 **–** 1.09 | .939 |
| Family support subscale * violence exposure |  |  |  |
| None^b^ | [reference] | | |
| Personal severe physical violence only | 0.97 | 0.78 **–** 1.21 | .787 |
| Both^c^ | 1.01 | 0.84 **–** 1.20 | .954 |
| Friend support subscale * violence exposure |  |  |  |
| None^b^ | [reference] | | |
| Personal severe physical violence only | 0.91 | 0.78 **–** 1.07 | .275 |
| Both^c^ | 1.10 | 0.94 **–** 1.28 | .237 |

*Note.* CI=confidence interval; OR=odds ratio. ^a^Adjusted simultaneously for biological sex, family history of psychopathology, and childhood emotional and behavioural problems (attention deficit and hyperactivity disorder, conduct disorder, symptoms of depression and anxiety, self-harm and suicide attempts, and psychotic symptoms). All models account for the non-independence of twin observations. ^b^Includes adolescents with no exposure to either personal severe physical violence or high levels of neighbourhood disorder, and those with exposure to high levels of neighbourhood disorder only. ^c^Includes adolescents with exposure to both personal severe physical violence and high neighbourhood disorder. N=1,903.

**References**

1. Trouton A, Spinath FM, Plomin R (2002) Twins early development study (TEDS): a multivariate, longitudinal genetic investigation of language, cognition and behavior problems in childhood. Twin Res 5:444-448. https://doi.org/10.1375/twin.5.5.444
2. Moffitt TE, E-Risk Study Team (2002) Teen-aged mothers in contemporary Britain. J Child Psychol Psychiatry 43:727–742. https://doi.org/10.1111/1469-7610.00082
3. CACI Information Services, 2006. ACORN user guide. CACI, London, UK.
4. Finkelhor D, Shattuck A, Turner HA, Ormrod R, Hamby SL (2011) Polyvictimization in developmental context. J Child Adolesc Trauma 4:291-300. <https://doi.org/10.1080/19361521.2011.610432>
5. Hamby S, Finkelhor D, Ormrod D, Turner H (2004) The comprehensive JVQ administration and scoring manual. Durham, NH: University of New Hampshire, Crimes Against Children Research Centre.
6. Fisher HL, Caspi A, Moffitt TE, Wertz J, Gray R, Newbury J, Ambler A, Zavos H, Danese A, Mill J, Odgers CL, Pariante C, Wong, CCY, Arseneault, L (2015) Measuring adolescents' exposure to victimization: the environmental risk (E-Risk) longitudinal twin study. Dev Psychopathol 27(4pt2):1399-1416. <https://doi.org/10.1017/S0954579415000838>
7. Caspi A, Moffitt TE, Thornton A, Freedman D, Amell JW, Harrington H, Smeijers J, Silva PA (1996) The life history calendar: A research and clinical assessment method for collecting retrospective event-history data. Int J Methods Psychiatr Res 6:101–114. [https://doi.org/10.1002/(SICI)1234-988X(199607)6:2<101::AID-MPR156>3.3.CO;2-E](https://psycnet.apa.org/doi/10.1002/(SICI)1234-988X(199607)6:2%3C101::AID-MPR156%3E3.3.CO;2-E)
8. American Psychiatric Association (2013) Diagnostic and Statistical Manual of Mental Disorders: DSM-5, 5th ed. American Psychiatric Association, Washington, DC
9. American Psychiatric Association (1994) Diagnostic and Statistical Manual of Mental Disorders: DSM-IV, 4th ed. American Psychiatric Association, Washington, DC
10. Heatherton TF, Kozlowski LT, Frecker RC, Fagerström KO (1991) The Fagerström Test for Nicotine Dependence: A revision of the Fagerström Tolerance Questionnaire. Br J Addict 86:1119-1127. <https://doi.org/10.1111/j.1360-0443.1991.tb01879.x>
11. Hill LS, Reid F, Morgan JF, Lacey JH (2010) SCOFF, the development of an eating disorder screening questionnaire. Int J Eat Disord 43:344-351. <https://doi.org/10.1002/eat.20679>.
12. Morgan JF, Reid F, Lacey JH (1999) The SCOFF questionnaire: Assessment of a new screening tool for eating disorders. BMJ 319:1467-1468. <https://doi.org/10.1136/bmj.319.7223.1467>
13. Polanczyk G, Moffitt TE, Arseneault L, Cannon M, Ambler A, Keefe RSE, Houts R, Odgers CL, Caspi A (2010) Etiological and clinical features of childhood psychotic symptoms: Results from a birth cohort. Arch Gen Psychiatry 67:328-338. <https://doi.org/10.1001/archgenpsychiatry.2010.14>.
14. March JS, Parker JD, Sullivan K, Stallings P, Conners CK (1997) The Multidimensional Anxiety Scale for Children (MASC): Factor structure, reliability, and validity. J Am Acad Child Adolesc Psychiatry 36:554–565. <https://doi.org/10.1097/00004583-199704000-00019>
15. Kovacs M (1992) Children's Depression Inventory (CDI) Manual. Multi-Health Systems, Toronto
16. Fisher HL, Moffitt TE, Houts RM, Belsky DW, Arseneault L, Caspi A (2012) Bullying victimisation and risk of self harm in early adolescence: Longitudinal cohort study. BMJ 344:e2683. https://doi.org/10.1136/bmj.e2683
17. Trzesniewski KH, Donnellan MB, Moffitt TE, Robins RW, Poulton R, Caspi A (2006) Low self-esteem during adolescence predicts poor health, criminal behavior, and limited economic prospects during adulthood. Dev Psychol 42:381-390. [https://doi.org/10.1037/0012-1649.42.2.381](https://psycnet.apa.org/doi/10.1037/0012-1649.42.2.381)
18. Weissman MM, Wickramaratne P, Adams P, et al. (2000) Brief screening for family psychiatric history: The family history screen. Arch Gen Psychiatry 57(7):675-682. https://doi.org/10-1001/pubs.Arch Gen Psychiatry-ISSN-0003-990x-57-7-yoa8214
19. Milne BJ, Caspi A, Crump R, Poulton R, Rutter M, Sears MR, Moffitt TE (2009) The validity of the family history screen for assessing family history of mental disorders. Am J Med Genet Part B 150:41–49. <https://doi.org/10.1002/ajmg.b.30764>
